# Overexpression of an exotic thermotolerant β-glucosidase in *trichoderma reesei* and its significant increase in cellulolytic activity and saccharification of barley straw

**DOI:** 10.1186/1475-2859-11-63

**Published:** 2012-05-20

**Authors:** Mehdi Dashtban, Wensheng Qin

**Affiliations:** 1 Biorefining Research Institute and Department of Biology, Lakehead University, 955 Oliver Road, Thunder Bay, Ontario, Canada, P7E 5E1

**Keywords:** Trichoderma reesei, Genetic engineering, β-glucosidase, Periconia sp.

## Abstract

**Background:**

*Trichoderma reesei is* a widely used industrial strain for cellulase production, but its low yield of β-glucosidase has prevented its industrial value. In the hydrolysis process of cellulolytic residues by *T. reesei*, a disaccharide known as cellobiose is produced and accumulates, which inhibits further cellulases production. This problem can be solved by adding β-glucosidase, which hydrolyzes cellobiose to glucose for fermentation. It is, therefore, of high vvalue to construct *T. reesei* strains which can produce sufficient β-glucosidase and other hydrolytic enzymes, especially when those enzymes are capable of tolerating extreme conditions such as high temperature and acidic or alkali pH.

**Results:**

We successfully engineered a thermostable β-glucosidase gene from the fungus *Periconia sp.* into the genome of *T. reesei* QM9414 strain. The engineered *T. reesei* strain showed about 10.5-fold (23.9 IU/mg) higher β-glucosidase activity compared to the parent strain (2.2 IU/mg) after 24 h of incubation. The transformants also showed very high total cellulase activity (about 39.0 FPU/mg) at 24 h of incubation whereas the parent strain almost did not show any total cellulase activity at 24 h of incubation. The recombinant β-glucosidase showed to be thermotolerant and remains fully active after two-hour incubation at temperatures as high as 60°C. Additionally, it showed to be active at a wide pH range and maintains about 88% of its maximal activity after four-hour incubation at 25°C in a pH range from 3.0 to 9.0. Enzymatic hydrolysis assay using untreated, NaOH, or Organosolv pretreated barley straw as well as microcrystalline cellulose showed that the transformed *T. reesei* strains released more reducing sugars compared to the parental strain.

**Conclusions:**

The recombinant *T. reesei* overexpressing *Periconia sp.* β-glucosidase in this study showed higher β-glucosidase and total cellulase activities within a shorter incubation time (24 h) as well as higher hydrolysis activity using biomass residues. These features suggest that the transformants can be used for β-glucosidase production as well as improving the biomass conversion using cellulases.

## Background

Lignocellulose, a renewable organic material, is the major structural component of plants. It is primarily composed of three major components: cellulose, hemicellulose, and lignin [1]. Large quantities of lignocellulosic wastes produced by different industries, such as the paper- making industry, are released to the environment on a daily basis causing a variety of environmental issues. Bioconversion of biomass is a promising solution to overcome some of the environmental issues associated with the lignocellulosic wastes as well as providing alternative energy resources such as bioethanol. Different microorganisms such as fungi and bacteria primarily initiate the bioconversion of lignocellulosic residues through a process known as hydrolysis. Fungi, however, have received the greatest interest due to their production of high quantities of extracellular cellulolytic enzymes. However, the disadvantage of the fungal system is that many natural fungal strains lack of some of lignocellulolytic enzymes necessary for efficient bioconversion processes [1,2]. Thus, the initial bioconversion of biomass into sugars remains a key bottleneck in the process of biofuel production. To this end new biotechnological solutions, such as genetic engineering of microorganisms, to improve lignocellulolytic enzymes efficiencies and enzymes able to tolerate harsh conditions are necessary [2].

The ascomycete *Hypocrea jecorina* (anamorph *Trichoderma reesei*) is one of the most studied and industrially important cellulolytic fungi. *T. reesei* is capable of efficiently degrading plant cell wall polysaccharides such as cellulose and hemicelluloses. *T. reesei* also produces a number of cellulases including cellobiohydrolases (exoglucanases) and endoglucanases as well as a set of hemicellulases and pectin degrading enzymes [2,3]. Additionally, a few different β- glucosidases (BGL) have been identified in *T. reesei*. These three groups of cellulolytic enzymes, i.e., exoglucanases, endoglucanases and β-glucosidases work efficiently on cellulolytic fibers in a synergistic manner. These β-glucosidases were reported to be extracellular [4], cell wall-bound [5], membrane-bound [6], and intracellular [7]. Most of *T. reesei* cellulases are inducible enzymes and their transcripts are not formed in the presence of monosaccharides in the growth medium. This means that an inducer is required in order for *T. reesei* to produce cellulases [3]. Cellulose polymer acts as a natural inducer despite the fact that it cannot transfer through the cell membrane due to its insolubility. Different investigations into the cellulases gene regulation in *T. reesei* have resulted in different proposed models, however, they all tend to agree that the actions of cellulases lead to the formation of a cellulase inducer [3]. The leading candidate for control of cellulolytic enzymes production in *T. reesei* is β-glucosidase, which is responsible for the production of an inducer, sophorose [3]. This was supported by Mach (1995), who disrupted the gene *cel3a* (*bgl1*) encoding the major extracellular β-glucosidases in *T. reesei* and demonstrateda delay in the induction of the other cellulases genes only by cellulose, but not in the presence of sophorose [3,8]. Thus, β-glucosidase production remains a key bottleneck in the process of cellulase production by *T. reesei*. Efforts have been made to improve cellulases production in *T. reesei* by homologues or heterologous overexpression of β-glucosidase genes [9-11]. Additionally, β-glucosidase production is important for its potential applications in many different industries such as food [12], winemaking [13] and textile [14].

An endophytic fungus *Periconia sp.* belonging to phylum Ascomycota was selected among 100 studied fungal strains by Harnpicharnchai et al. (2009) due to its highest β-glucosidase activity at elevated temperatures [15]. They ultimately identified the gene encoding β-glucosidase (*bgl1*), cloned and expressed it in *Pichia pastoris*. The purified protein was reported to have high β-glucosidase activity at higher temperatures, and was also active at a wide pH range [15]. Thermostable β-glucosidases with high enzyme activity are especially useful for the bioconversion of lignocellulosic residues at elevated temperature [1].

In the current study, we overexpressed the β-glucosidase gene (*bgl1*) from *Periconia sp.* in *T. reesei* QM9414 under the control of a promoter region of *T. reesei tef1α* (encoding translation elongation factor 1-alpha). The β-glucosidase production and total cellulase activity of the BGLI-overexpressing transformants were significantly increased. Additionally, hydrolysis efficiency of the BGLI-overexpressing transformants was also significantly increased using NaOH- or Organosolv-pretreated barley straw.

## Results and Discussion

### Overexpression of extracellular β-glucosidase from *periconia sp.* In *T. Reesei* QM9414

To increase the production of β-glucosidase, and ultimately the overall cellulolytic ability of *T. reesei*, the *bgl1* gene encoding an extracellular β-glucosidase (BGLI) in *Periconia sp*. was selected. This gene was selected as it has been previously shown to produce a thermotolerent β- glucosidase [15]. Ultimately, *bgl1* was isolated by Harnpicharnchai et al. (2009) from the strain and subsequently cloned into *P. pastoris*[15]. The optimal temperature and pH for the enzymen was reported to be 70°C and 5.0-6.0, respectively. The enzyme retained 60% of its activity at 70°C after 1.5 h incubation. Moreover, the enzyme remained fully active when incubated for 2 h at pH ≥ 6.0 [15]. BGLI belongs to family 3 glycoside hydrolases (EC 3.2.1.21) and as the other members of the group showed high activity toward aryl β-*D*-glucosides, cellobiose, and cellooligosaccharides [15,16]. Finally, the addition of BGLI to a commercial cellulase (Celluclast ® 1.5 L) improved the hydrolysis rate of rice straw into simple sugars [15]. These attractive features made the BGLI as the candidate for overexpression of the gene and thus improvement of β-glucosidase activity in *T. reesei*.

**Figure 1:**
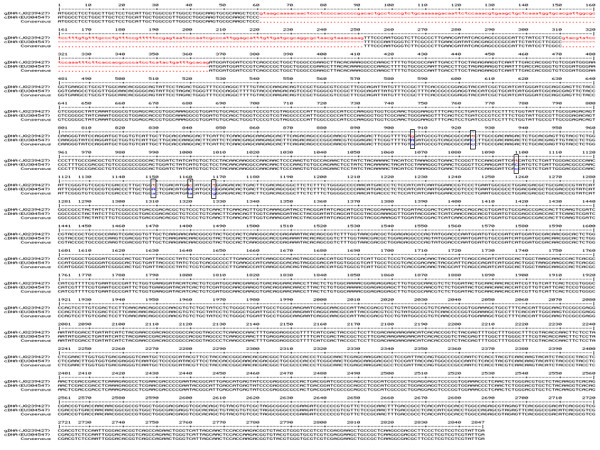
**Alignment of*Periconia sp. bgl1*genomic DNA and cDNA using MultAlin software.** Intron regions are shown in lower case in red while the coding regions are shown in upper case and black. The 6 single-nucleotide differences between the genomic DNA (Accession No. JQ239427) and previously reported cDNA (Accession No. EU304547) (including: A660G, C678T, C851T, A903T, A914C and G921T) are shown in lower case and highlighted in boxes.

In our study, *Periconia sp.* total RNA was extracted and cDNA was constructed. The 2601 bp *bgl1* gene was amplified through PCR using Full-Beta primers (Table 1) designed according to cDNA sequence of *Periconia sp bgl1* reported by Harnpicharnchai et al. (Accession No. EU304547). However, our cDNA sequencing results showed that our cloned *bgl1* sequence has 6 nucleotide differences compared to the reported cDNA sequence. To confirm the result, *Periconia sp. bgl1* genomic DNA was also cloned and sequenced. The sequencing results using the genomic DNA confirmed the 6 nucleotide difference. These nucleotides were included A660G, C678T, C851T, A903T, A914C and G921T (numbers are based on the cDNA sequences). Four of the six variations made changes in the codon, but coded the same amino acids (aa 220; A660G, aa226; C678T, aa301; A903T and aa307; G921T). The other two however, coded different amino acids (C851T: T → I, and A914C: N → T, aa 284 and aa 305, respectively). The 2847 bp *bgl1* genomic DNA contained three exons (1–60, 252–310 and 366–2847) and two introns (61–191 and 180–250) was submitted to the GenBank [Accession No. JQ239427]. *Periconia sp. bgl1* genomic DNA and cDNA were aligned using MultAlin software (multiple sequence alignment by Florence Corpet) and presented in Figure 1[17].

**Table 1:** List of oligonucleotides used in the study for the genes amplification (restriction sites are underlined)

**Primers set/gene**	**Primer name**	**Sequence (**5'-3')	**Expected fragment size (bp)**
***bgl1***	Full-Beta-F	ATGGCCTCCTGGCTTGCT	2601
	Full-Beta-R	TCAATACGACGACGAGGGAA	
***cbh1***	*cbh1*-F	AGGTCACCTTCTCCAACATC	573
	*cbh1*-R	AGAGCGGCGATTCTACGGGT	
	***F1***	AGATCGATATGGCCTCCTGGCTTGCTCCTG	
***bgl1-cbh1*fusion**	Fuse-REV1	AGAAGGTGACCTTCAATACGACGACGAGGGAA	3187
	Fuse- F2	GTCGTATTGAAGGTCACCTTCTCCAACATC	
	REV2	GGATCGATAGAGCGGCGATTCTACGGGTTA	
**In-Fusion**	In-Fusion-F	CACAAACCGTCATCGATATGGCCTCCTGGCTTGCT	3205
	In-Fusion-R	TGCAGGTCGACATCGATAGAGCGGCGATTCTACGGGTTA	
***bgl1***	*bgl1*-Realtime-F	GCCATTCTATCCTTCGCC	154
	*bgl1*-Realtime-R	TGCCAACGCAGGCTTCAC	
***tef1a***	*tef1a*-Realtime-F	ACCAAGGCTGGCAAGTTC	162
	*tef1a* -Realtime-R	GACACCAGTCTCGATACG	

**Figure 2:**
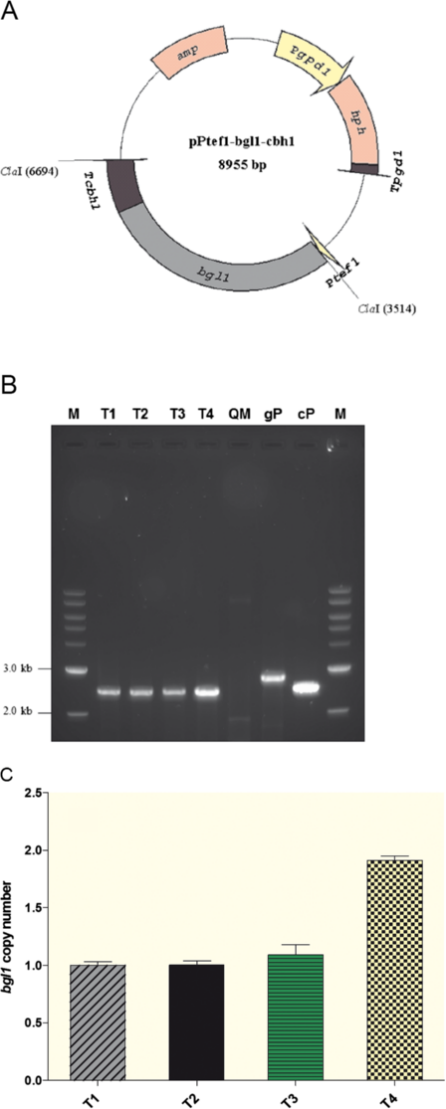
**Construction of BGLI-overexpression*T. reesei*strain.(A)** The structure of pPtef1- *bgl1-cbh1* expression vector using SimVector software. Ptef1 represents *tef1* promoter; *bgl1* represents β-glucosidase gene from *Periconia sp.*; T*cbh1* indicates the *cbh1* terminator; P*gpd1* represents *gpd1* promoter (glyceraldehyde-3-phosphate dehydrogenase gene); *hph* represents hygromycin B phosphotransferase gene; T*gpd1* indicates the *gpd1* terminator. **(B)** PCR assay ofthe *bgl1* gene in the selected overexpression transformants using full-bgl1 primers. All transformants (T1-T4) showed a 2601 bp band whereas the parent strain (QM) did not amplified any specific PCR products. *Periconia sp.* genomic DNA (gP) as well as cDNA (cP) were used as the positive controls which amplified 2847 and 2601 bp products, respectively. **(C)** qPCR analysis of the isolated genomic DNA from the transformants T1-T4 using Real-Time *bgl1* and *tef1a* primers. The transformants T1-T3 received one copy of the *bgl1* gene whereas T4 obtained two copies.

The *T. reesei* cellobiohydrolases I terminator region was used as the terminator to generate the *bgl1-cbh1* expression cassette. The *bgl1-cbh1* expression cassette was cloned into pPtef1 expression vector under the promoter regions of highly expressed gene *tef1α* to generate pPtef1*-bgl1-cbh1* (Figure 2A) and then transformed into the *T. reesei* QM9414. The plasmid contains hygromycin B phosphotransferase (*hph*) expression cassette which was used as the selection marker. Four mitotic stable hygromycin-resistant transformants were obtained and were selected for the single spore isolation to insure for the pure culture. The four transformants (named T1-T4) were cultured for more than 6 generations and *bgl1* was confirmed to be in the genome of the transformants via PCR and DNA sequencing (Figure 2B). The copy number of the *bgl1* expression cassette integrated into the *T. reesei* genome of the transformants was analyzed by quantitative Real-Time PCR (qRT-PCR) (Figure 2C). The result showed that three of the four transformants (T1, T2 and T3) obtained only one copy of the *bgl1* cassette whereas T4 received two copies.

**Figure 3:**
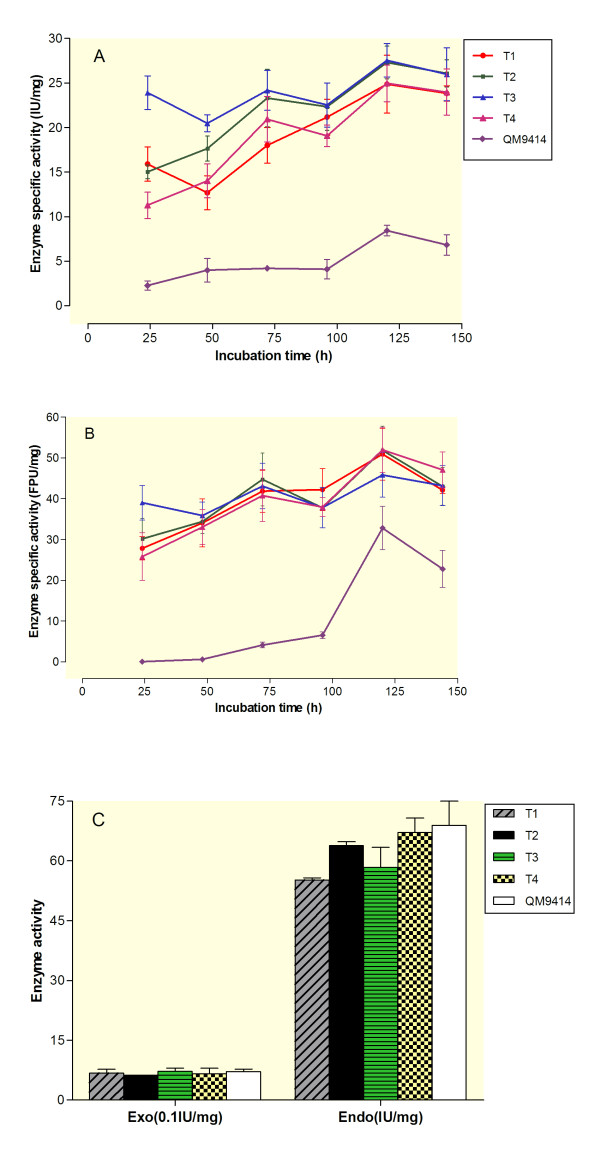
**The enzyme activities and time course study of BGLI-overexpressing transformants (T1-T4) and the parental strain (*T. reesei*QM9414).** The strains were pre- grown on MA-medium containing 1% glucose for 24 h and then mycelia were washed and grown for 144 h on MA-medium containing 1% microcrystalline for the induction. Enzyme activities were expressed as specific activities using international units per mg protein in the supernatant. **(A)** The β-glucosidase activity was measured every 24 h using the culture supernatant as the enzyme sources. The enzyme was incubated at 70°C for 10 min, pH 5.0 using 50 mM sodium citrate buffer. **(B)** Filter paper activity (FPA) was measured every 24 h using the culture supernatant and incubated at 50°C for 60 min, pH 4.8 using 75 mM citrate buffer. **(C)** Exoglucanases (Exo), and endoglucanases activities (EG) of the strains were measured after being induced with 1% microcrystalline cellulose for 120 h. No significant difference was obtained for Exo and EG activities of the four transformants compared to the parent strain usingone-way ANOVA at a confidence level of 99% (α = 0.01). Data are represented as the mean of three independent experiments and error bars denote standard error of the mean.

To test the β-glucosidase activity of the BGLI-overexpressing *T. reesei* transformants (T1-T4), they were cultivated in a microcrystalline cellulose containing medium as the cellulase inducer for 144 h. The β-glucosidase activity of the transformants was measured using the culture supernatant every 24 h and was compared to the parent strain (*T. reesei* QM9414) (Figure 3A). All the transformants showed very high β-glucosidase activity compared to the parent strain over the time course study. High β-glucosidase activity in the transformants was seen at the first time point (24 h) after the induction with about 23.9 IU/mg for T3 which is 10.5-fold higher than the parental β-glucosidase activity (Figure 3A). The β-glucosidase activity of the transformants and the parent strain slowly increased over the rest of the time points with a peak at 120 h post induction. The maximum activity was seen in two transformants (T2 and T3) at about 27 IU/mg at 120 h. The parent strain also showed the maximum activity at 120 h with about 8.4 IU/mg, which is 3.2-fold lower than the maximum activity obtained for the transformants (T2 and T3). Although the transformant T4 received two copies of the *bgl1* expression cassette, our results showed that its β-glucosidase activity resembled the same as the other transformants with the single integrated expression cassette (Figure 3A). Levels of transcription and recombinant protein production in fungal transformants are mainly correlated with two factors including the gene copy number and the loci of the integration [18]. However, it has been reported that in some cases, high copy number transformants did not produce more recombinant protein in *T. reesei*[9,18,19]. This can be possibly due to different loci integration of the expression cassette in *T. reesei* genome. Improvement of β-glucosidase activity of *T. reesei* strains was previously reported by different groups where the activity was increased by 7.5 or 3.7-fold compared to the parent strain after 168 or 36 h incubation time, respectively [9,10]. Our results indicated that the activity of our constructed transformants is higher than (10.5-fold) previous studies. In addition, the transformants showed high β-glucosidase activity within a shorter induction time (24 h). Our recombinant strain (T3) showed β-glucosidase activity of 23.9 IU/mg after the first 24 h of incubation which is 2.8-fold higher than the maximal β-glucosidase activity of the parent strain (8.4 IU/mg) after 120 h of incubation. Higher β-glucosidase production, as well as shorter incubation time, suggests that our transformants can be used for their potential industrial application for the production of β-glucosidase.

Despite *Aspergillus niger, T. reesei* produces very low amounts of BGL causing the accumulation of cellobiose as the end product and thus inhibits further cellulose hydrolysis [1,20]. Thus, it has been suggested that by improving the BGL activity of *T. reesei* its cellulose hydrolysis activity would also improve. Different studies have shown that overexpression of different BGL in different *T. reesei* strains results in higher cellulolytic activity. For example, total cellulase activity of different recombinant *T. reesei* strains such as RUT-C30 or PC-3-7 were improved by 2.3 or 1.3-fold respectively, when compared to their parent strains [9,10]. Our four transformants (T1-T4) were grown in a cellulase-inducing medium (MA-medium containing microcrystalline cellulose) for 144 h and their total cellulase activity (or filter paper assay, FPA) were compared to the parent strain every 24 h (Figure 3B). The maximum FPA activity was seen for T4 with about 51.9 FPU/mg activity after 120 h of incubation. The parent strain also reached its maximum FPA activity (about 32.8 FPU/mg) at 120 h, which is 1.58-fold less compared to the activity obtained for T4. FPA activity of the parent strain was very low over the first 48 h of the incubation whereas high FPA activity was obtained for all the four transformants after the first 24 h of incubation (Figure 3B). For example, T3 showed high FPA activity with about 39.0 FPU/mg at 24 h of incubation which is 1.18-fold higher than the maximal FPA activity of the parent strain (about 32.8 FPU/mg) obtained after 120 h of incubation. Thus, all the four transformants showed higher FPA activity within a shorter incubation time compared to the parent strain (Figure 3B).

Cellobiohydrolase (exoglucanases, Exo) and endoglucanases (EG) activities of BGLI- overexpressing transformants and the parental strain were also measured at 120 h, as this is where the maximal BGL and FPA activities were seen (Figure 3C). All the four transformants showed similar level of Exo activity compared to the parent strain. Although T2 and T4 showed slight decreased in Exo activity compared to the parent strain but no significant difference was obtained (ANOVA, P < 0.01). This may also be a result of different loci integration of the BGLI- overexpressing transformants. Similar result was also reported by Zhang study (2010) in which the Exo activity of some of the overexpressing transformants were lower than that of the parent *T. reesei* strain [9]. In the case of EG activity, however, transformants T1-T3 showed lower level of EG activity whereas the transformant T4 showed almost the same activity compared to the parent strain (Figure 3C). However, no significant difference in EG activity was obtained between the four transformants and the parent strain (ANOVA, P < 0.01). The total cellulase activity of our transformants was significantly higher than the parent strain despite the fact that btheir Exo and EG activities in two of the four transformants were slightly decreased. This supports the hypothesis that the lower β-glucosidase production by *T. reesei* causes the inhibition of further hydrolysis of cellulosic residues to the end product [1].

### Characterization of the BGLI-overexpressing transformants

**Figure 4:**
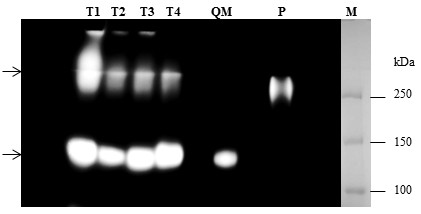
**Identification of β-glucosidase in BGLI-overexpressing transformants (T1-T4) by MUG-zymogram assay.** Proteins (culture supernatant) from BGLI-overexpressing transformants (T1-T4) and the parental strain (*T. reesei* QM9414, lane QM) were separated in 8% native PAGE. Culture supernatant of *Periconia sp.* (*bgl1* donor strain) (lane P) grown on MA-medium containing 1% microcrystalline was used as the positive control. β-glucosidase activity was detected by MUG-zymogram assay. Lower arrow indicates the native extracellular β-glucosidase found in *T. reesei* strain (lanes T1-T4 and QM) whereas the upper arrow represents active β-glucosidase correlated to *Periconia sp.* BGLI only found in the BGLI- overexpressing transformants as well as *Periconia sp.* (lanes T1-T4 and P). Lane M, molecular weight marker.

Identification of β-glucosidase BGLI-overexpressing transformants (T1-T4) was done using 4-methylumbelliferyl β-D-glucopyranoside (MUG) in a MUG-zymogram assay (Figure 4). Proteins from the culture supernatant were first separated in an 8% native PAGE gel, with β- glucosidase activity ultimately detected using a MUG-zymogram assay (Figure 4). One enzymatically active protein was observed in the culture supernatant of both the BGLI- overexpressing strains as well as the parent strain *T. reesei* QM9414 (Figure 4, lanes T1-T4 and QM). This β-glucosidase activity has been reported as the native extracellular β-glucosidase in *T. reesei* strain (BGLI) with a molecular weight about 75 kDa [8]. However, a second active β- glucosidase with higher molecular weight was only obtained from the transformants, which is correlated to the recombinant BGLI (Figure 4, lanes T1-T4). A sample from the culture supernatant of *Periconia sp.* (*bgl1* donor) was also compared to confirm the presence of the recombinant BGLI in the BGLI-overexpressing transformants (Figure 4, lane P). Harnpicharnchai et al. (2009) also reported that the *Periconia sp.* BGLI showed a higher molecular weight band on the native gel (> 150 kDa) although its molecular weight predicted to be around 95 kDa [15]. This study also showed that overexpressed *Periconia* sp. BGLI (in *P. pastoris*) migrates slowly on a SDS-PAGE gel with a molecular weight around 130 kDa [15]. They proposed that the slower migration is due to the protein glycosylation (BGLI has 16 putative *N*-glycosylation sites predicted by PROSITE) which was also confirmed by the experimental approaches [15].

**Figure 5:**
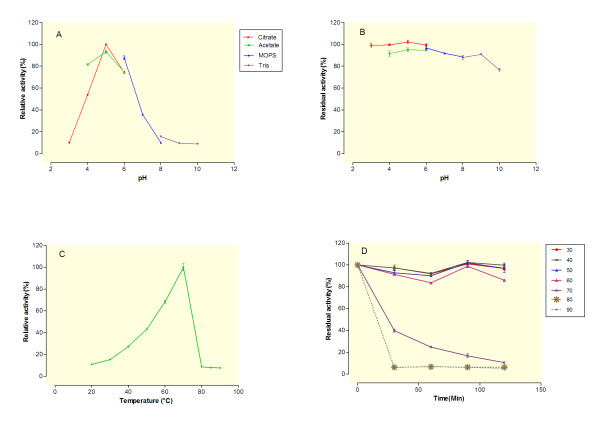
**Effect of pH and temperature on β-glucosidase activity of the selected BGLI- overexpressing transformant (T3).(A)** pH profile was determined by incubating the enzyme (culture supernatant) at 70°C for 10 min at different pH, using 50 mM sodium citrate (pH 3.0- 6.0), sodium acetate (pH 4.0-6.0), MOPs (pH 6.0-8.0) and Tris buffer (pH 8.0-10.0). **(B)** The remaining β-glucosidase activity was determined (at 70°C in 50 mM sodium citrate buffer, pH 5.0 for 10 min) after incubating the enzyme at 25°C for 4 h at the different pHs. **(C)** Temperature profile was determined by incubating the enzyme (culture supernatant) in 50 mM sodium citrate buffer (pH 5.0) for 10 min at different temperature (30–90°C). **(D)** Thermal stability was carried out by incubating the enzyme in 50 mM sodium citrate buffer (pH 5.0) at different temperature (30–90°C) for 30, 60, 90 and 120 min before the remaining activity was assayed (at 70°C in 50 mM sodium citrate buffer, pH 5.0 for 10 min).

The effect of pH on β-glucosidase activity of BGLI-overexpressing transformants was determined using *p*-nitrophenyl-β-D-glucopyranoside (*p*NPG) as the substrate. The culture supernatant of the selected transformant T3 (based on its β-glucosidase activity) was incubated at 70°C for 10 min at different pH (3.0-10.0). Although, the maximal enzyme activity was obtained at pH 5.0, the enzyme showed over 80% activity in a pH range from 4.0 to 6.0 (Figure 5A). Harnpicharnchai and et al. (2009) reported similar results using purified *Periconia sp.* BGLI overexpressed in *P. pastoris* expression system [15]. For the determination of enzyme stability at different pH, the culture supernatant was incubated in the various pH buffers (3.0-10.0) for 4 h at 25°C before determining the remaining β-glucosidase activity (Figure 5B). Our results indicated that the β-glucosidase remain over 88% active in a pH range from 3.0 to 9.0. However, the remaining activity decreased to about 76% when the pH increased to 10.0 (Figure 5B). Similar results were obtained for the pH ranges from 6.0-10.0 by the Harnpicharnchai study using the purified BGLI enzyme overexpressed in *P. pastoris*[15]. Nazir et al. (2009), also reported an active β-glucosidase (BGI) from *Aspergillus terreus* after 4 h of incubation under a broad range of pH (5.0-10.0) [21].In another study by Ng et al. (2010) pH stability of the crude β- glucosidase from *Penicillium citrinum* YS40-5 was tested over a wide pH range from 1.0-10.0 after 24 h incubation at 4°C [22]. Surprisingly, the enzyme retained over 85% of its maximal activity over the entire pH range [22]. Since citrate buffer at pH 5.0 gave the highest level of enzyme activity, it was chosen to be used as the standard assay condition in the all following experiments when the BGLI activity was measured.

The temperature influence on β-glucosidase activity of BGLI-overexpressing transformants was determined by monitoring the hydrolysis of *p*NPG at 20–90°C (Figure 5C). The maximal enzyme activity obtained at 70°C but it exhibited more than 80% of the maximal activity between 65 and 75°C. This is in accordance to the reported optimal enzyme temperature using pure *Periconia sp.* BGLI overexpressed in *P. pastoris* expression system [15] and also it is in the range of reported optimal temperature for different β-glucosidase (45–75°C) [1]. For example, the optimal temperature of a thermotolerant β-glucosidase from moderately thermophilic fungus *Talaromyces emersonii* also overexpressed in *T. reesei* was determined to be at 71.5°C [23]. The thermal stability of BGLI was tested by incubating the enzyme at different temperature (30–90°C) for 30–120 min (Figure 5D). The residual activity was measured every 30 min at the optimal pH and temperature (pH 5.0 and 70°C). The enzyme maintained over 85% of its maximal activity after 2 h incubation at different temperatures up to 60°C. The enzyme activity, however, decreased to about 40% of its maximal activity after 30 min of incubation at 70°C. Higher temperatures (≥80°C) inactivated the enzyme and only about 5% of the maximal activity was maintained after 30 min of incubation. Thermotolerant β-glucosidase from *T. emersonii* was reported to maintain 50% of its maximal activity at 65°C after 62 min of incubation [23].

Our overexpressed BGLI also maintained its maximal activity after four months incubation at 4°C (data not shown here). Overall, our BGLI-overexpressing transformants showed very high β-glucosidase activity which can tolerate a wide range of pH (3.0-10.0), high temperature (up to 60°C) and also remain fully active after long time storage at 4°C in the absence of any stabilizer. These unique features suggest that our BGLI-overexpressing transformants are superior candidates for their potential biotechnological applications. Many recent bioconversion research studies focused on two main strategies including enhancing overall fungal hydrolytic activities as well as identifying stable enzymes able to function under harsh conditions [1]. Acidic pretreatment of lignocellulosic residues seems a preferred option due to fungal cellulolytic enzymes activity at lower pH (usually 4.0-5.0). Enzymes able to remain active at higher temperatures and also retain their activity at lower pH values are more\ suitable for pretreatment methods where acid and high temperatures are applied [1]. The overall hydrolytic activity can be enhanced by thermostable enzymes through their higher specific activity and higher stability [1]. β-glucosidases able to tolerate harsh conditions (i.e. acidic and/or basic pH conditions as well as high temperatures) have great potential biotechnological applications in different industries such as food [12], wine [13] and textile production [14]. Thermotolerant β-glucosidases, for example, represent valuable characteristics such as reduction of the risks associated with microbial contamination in the process as well as substrate viscosity which leads to higher reaction velocities and thus improved hydrolysis efficiency.

### Enzymatic hydrolysis of biomass by the BGLI-overexpressing *T. Reesei* transformants

**Figure 6:**
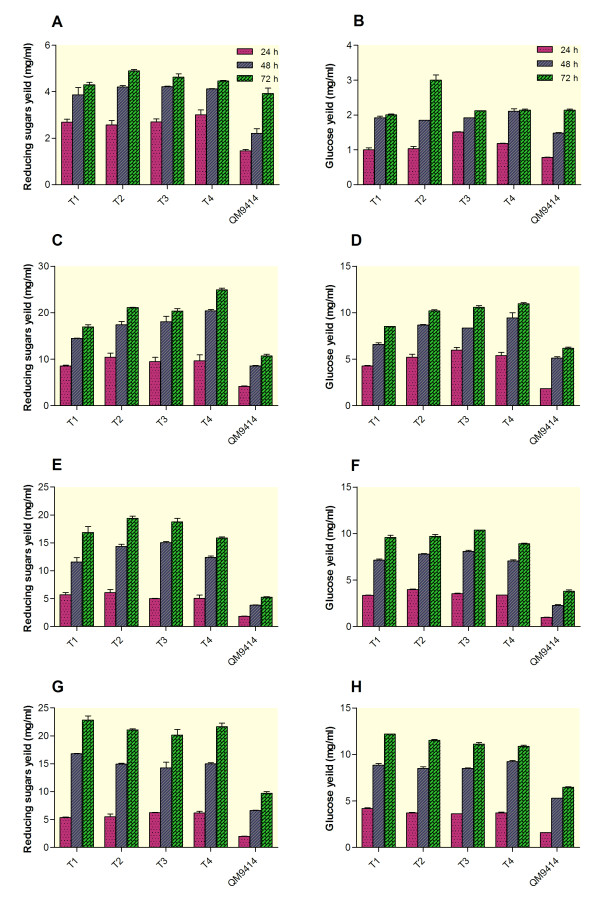
**Enzymatic hydrolysis of barley straw and microcrystalline cellulose by BGLI- overexpressing transformants (T1-T4) and the parental strain (*T. reesei*QM9414).(A** and **B)** Reducing sugars and glucose yield (mg/mL) released using untreated barley straw, respectively. **(C** and **D)** Reducing sugars and glucose yield (mg/mL) released using NaOH- pretreated barley straw, respectively. **(E** and **F)** Reducing sugars and glucose yield (mg/mL) released using Organosolv-pretreated barley straw, respectively. **(G** and **H)** Reducing sugars and glucose yield (mg/mL) released using microcrystalline cellulose, respectively. 750 μl of the culture supernatants (as the enzyme source) were added to tubes containing 750 μl of 50 mM sodium citrate buffer (pH 5.0) and 0.045 g (3%) either barley straw or microcrystalline cellulose. The tubes were incubated at 50°C for 72 h and reducing sugars as well as glucose released were measured every 24 h. Data are represented as the mean of three independent experiments and error bars denote standard error of the mean. Total protein loading was as follow; T1: 0.22 mg; T2: 0.26 mg; T3: 0.24 mg; T4: 0.19 mg, and QM9414: 0.22 mg.

Efficient hydrolysis of cellulose and hemicelluloses of lignocellulosic residues to their soluble monomeric sugars is an important step in bioconversion process. For this reason, much effort has been done to obtain efficient microorganisms with robust lignocellulolytic activities [1]. To this end, the hydrolysis efficiency of the BGLI-overexpressing *T. reesei* transformants using barley straw was investigated (Figure 6A-H).

For the untreated barley straw (Figure 6A and B), all the transformants released higher amount of reducing sugars (RS) (maximum 4.21 mg/mL for T3) and glucose (G) (maximum 1.92 mg/mL for T3) which were 90 and 30% higher than the parent strain (2.21 mg/mL RS and 1.48 mg/mL G) over the first 48 h of hydrolysis incubation time, respectively. Longer incubation time (72 h) slightly increased the RS and G released by the transformants (maximum for T2 with 4.90 and 3.0 mg/mL RS and G, respectively). In the case of the parental strain, the longer incubation time (72 h) also improved the hydrolysis rate and the level of RS and G was 3.92 and 2.14 mg/mL, respectively. This was 25 and 40% lower than the maximal RS and G productions by the transformant T2. This suggest that in the absence of any pretreatment, our BGLI-overexpressing transformants efficiently hydrolyze barley straw within a shorter time compared to the parent strain.

Hydrolysis of barley straw treated with NaOH was also tested using all four transformants and the parental strain (Figure 6C and D). The results indicated that after 72 h, all four transformants released higher amount of RS and G (maximum 24.96 and 11.0 mg/mL for T4, respectively), which were 2.33- and 1.77-fold higher than the enzymatic hydrolysis by the parent strain (10.71 and 6.18 mg/mL, respectively). Similar results were also observed when Organosolv-treated barley straw was used (Figure 6E and F). After 72 h of the incubation time, the maximal RS and G released was obtained for T2 (19.42 and 9.71 mg/mL, respectively), which were 3.67- and 2.55-fold higher than the parent strain (5.28 and 3.80 mg/mL, respectively). Hydrolysis of microcrystalline cellulose was also tested using all four transformants as well as the parental strain (Figure 6G and H). After 72 h of the incubation time, all four transformants released higher amount of RS and G (maximum 22.80 and 12.20 mg/mL for T1, respectively), which were 2.34- and 1.89-fold higher than the enzymatic hydrolysis by the parent strain (9.71 and 6.45 mg/mL, respectively). Our results demonstrated that the BGLI- overexpressing transformants produced more efficient enzymes which facilitate hydrolysis of the cellulosic substrates. This may be a direct result of removing β glucosidase limitation on further cellulose hydrolysis [1].

## Conclusion

In our study, a thermotolerant β-glucosidase (BGLI) from *Periconia sp.* was overexpressed in *T. reesei* QM9414. The β-glucosidase activity and total cellulase activity of the recombinant *T. reesei* strains overexpressing BGLI are significantly increased. The BGLI- overexpressing transformants showed higher biomass hydrolytic efficiency, suggesting that they can be used in the hydrolysis step in biomass conversion. High β-glucosidase activity, wide pH range tolerant and high temperature resistance makes the transformants excellent candidates for their potential application for the production of β-glucosidase as well as improving the biomass conversion using cellulases.

## Methods

### Chemicals

All the chemicals and reagents were of analytical grade. Microcrystalline cellulose was obtained from J. T. Baker (Phillipsburg, NJ, U.S.A.). Barley straw (obtained from Gammondale Farm, Thunder Bay, Canada) was grounded in a Wiley mill and then sieved to less than 20 mesh and dried in an oven to a constant weight at 70°C before use.

### Pretreatment of barley straw

Organosolv pretreatment of barley straw was done (in Dr. Charles Xu’s lab, Lakehead University, Canada) using a 1 L autoclave reactor (Autoclave Engineers, U.S.A.) [24]. Briefly, 50 g of previously grounded barley straw was used as the feedstock. The feedstock/solvent ratio was 1:10 (w/v) and 50% ethanol: water (v/v) was used as the solvent. The pressure was maintained at about 300 psi using nitrogen, and temperature was kept at 190°C. The reaction was mixed at 130 rpm and maintained for 4 h. Pretreated barley straw was subjected to a wash using 100% acetone. Following this, lignin was removed using filtration and cellulose and hemicellulose were obtained as solid residues. Solid residues (SR) were dried at 105°C overnight before weighing. The gaseous product inside the reactor was collected into a pre- vacuum fixed-volume (2800 mL) gas-collecting vessel. Liquefied lignin was subjected to a rotary evaporation under reduced pressure at 40°C to remove acetone and ethanol. The weight of the dry lignin was measured in order to estimate the lignin percentage. Yields of lignin and SR were calculated by the wt% of the mass of each product to the mass of the dry feedstock loaded into the reactor. Using this method, the lignin and SR content of barley straw were 8.77 (17.54%) and 27.46 (54.99%) g/50 g barley straw, respectively. The aqueous and gaseous products of barley straw were 13.77 (27.54%) g/50 g barley straw.

Alkali treatment was done using 2 g (2% w/v) of previously grounded barley straw according to the method described by Deshpande [25]. Briefly, the grounded barley straw was subjected to alkali treatment by soaking in NaOH solution at 2% (w/v) for 48 h at ambient temperature. The material was then washed thrice with water. The water for the fourth wash has 1% phosphoric acid added. The materials were subjected to two more washes with water and then dried to constant weight at 70°C.

For the estimation of various component of barley straw, sequential fractionation was carried out according to the method described by Arora and Sharma (2009) [26]. Briefly, one g of barley straw was suspended in 100 mL distilled water and kept at 100°C for 2 h in a water bath. The slurry was filtered and the residue was dried at 80°C to constant weight. The weight loss was considered as water soluble part (Table 2). To estimate hemicellulose and cellulose in barley straw, they were removed according to TAPPI T222 protocol with some modifications as described by Sharma and Arora (2010) [27,28]. Dried sample was briefly treated with 100 mL of 0.5 M H_2_SO_4_ at 100°C for 2 h. The content was filtered, dried, weighted and the loss considered as hemicellulose content (Table 2). Following this, the dried sample was treated with 40 mL 72% H_2_SO_4_ at 30°C for 1 h at 200 rpm. Subsequently, it was diluted to 3% concentration of H_2_SO_4_ with distilled water and autoclaved at 121°C for 45 min. The content was filtered, dried, weighed and the loss counted as cellulose content. Acid-soluble lignin was determined by measuring the absorbance of the supernatant at 205 nm according to TAPPI Useful Method UM 250 [29]. Finally, the acid-insoluble lignin content was measured after the weight of ash was measured by burning the samples in a muffle furnace at 525°C, according to TAPPI T211 protocol “Ash in wood, pulp, paper and paperboard: combustion at 525°C” [28] (Table 2).

**Table 2:** Composition of barley straw used in the study

	**Soluble part**	**Hemicellulose**	**Cellulose**	**Acid-soluble lignin**	**Acid-insoluble lignin**	**Ash**
**Untreated barley straw (%)**	13.13 ± 0.40	26.46 ± 3.67	32.60 ± 4.10	1.49 ± 0.53	24.01 ± 4.79	1.03 ± 0.06

Data are represented as the mean of three independent experiments and error denote standard error of the mean.

### Microorganism strains and culture conditions

*Escherichia coli* JM109 was used for vector construction and propagation. Endophytic fungus *Periconia sp.* (BCC2871, obtained from BIOTEC Culture Collection, Thailand) was used as the *bgl1* gene provider. Cellulase hyperproducing mutant *T. reesei* QM9414 (ATCC 26921, kindly provided by Dr. Tianhong Wang, Shandong University, China) was used as a host for the overexpression of *bgl1* gene. The fungal strains were grown and maintained on potato dextrose agar (PDA) containing 15.0 g/L starch, 20.0 g/L *D*-glucose, and 18.0 g/L agar [30]. PDA medium supplemented with 50 μg/mL hygromycin was used as a selection marker for screening of *T. reesei* transformants. Strains were grown in 250 mL flasks, on a rotary shaker (200 rpm) at 30°C, and in 50 mL of medium described by Mandel and Andreotti (MA-medium) [31] with the respective carbon source at a final concentration of 1% (w/v). The media containing the respective carbon sources were autoclaved at 121°C (15 lb psi) for 15 min.

### Construction of *bgl1* expression cassette

*Periconia sp.* total RNA was extracted using Ambion RNA extraction kit (Invitrogen, Canada) and cDNA was constructed using Fermentas first strand cDNA synthesis kit (Fermentas, Canada). The 2601 bp *bgl1* gene was amplified with PCR using Full-Beta primers (Table 1) designed according to cDNA sequence of *Periconia sp bgl1* (Accession No. EU304547) [15]. The *T. reesei* cellobiohydrolases I (cbh1) terminator region was used as the terminator. The 573 bp *cbh1* terminator was amplified by PCR using *cbh1* primers (Table 1) and *T. reesei* QM9414 chromosomal DNA as the template. The *bgl1* and *cbh1* PCR products were used as the templates to fuse the *cbh1* terminator to the 3′ end of *bgl1* gene (to generate *bgl1- cbh1* cassette) through fusion PCR using *bgl1-cbh1* fusion primers (Table 1). The fused PCR product (*bgl1-cbh1* cassette) was gel extracted and used as a template for In-Fusion cloning (In- Fusion primers, Table 1) into ClaI linearized pPtef1-hph vector (kindly provided by Dr. B. Seiboth, Vienna University of Technology, Austria) [32] using In-Fusion® Advantage PCR Cloning Kit (Clontech Laboratories, Inc., USA) to generate pPtef1-*bgl1-cbh1*. The plasmid carrying *bgl1-cbh1* cassette was transformed to *E. coli* and ampicillin was used to screen the transformant. The positive transformants were selected and inserted *bgl1-cbh1* cassette into the plasmid was confirmed using DNA sequencing.

### Transformation of *T. Reesei* QM9414 and molecular analysis of the transformants

*T. reesei* QM9414 protoplasts were prepared according to Szewczyk [33]. The protoplasts were then transformed with pPtef1-*bgl1-cbh1* containing hygromycin B phosphotransferase (*hph*) expression cassette as the selection marker, according to the method described by Szewczyk [33]. The transformants were screened on PDA plate containing 50 μg/mL hygromycin as the selection marker. Single spore separation was done to ensure a pure culture. The integration of pPtef1-*bgl1-cbh1* into the genome of *T. reesei* QM9414 was confirmed using full size *Periconia sp. bgl1* primers (Table 1), with an expected fragment length of 2.6 kb. To identify the gene copy number in the obtained positive transformants, qRT-PCR was carried out using extracted genomic DNA as the template and Real-Time primers (*bgl1* and *tef1a* Real-Time primers, Table 1) according to method described by Solomon [34]. *Tef1α* (translation elongation factor 1-alpha) was used to represent single copy region within the *T. reesei* genome (2279 bp in scaffold 6, from 764792–767070) confirmed by blasting *tef1α* sequence [GenBank: Z23012.1] [35] against *T. reesei* genome sequence using the *T. reesei* genome database v2.0 [36]. RT-PCRs were performed using a Bio-Rad CFXTM 96 Real-Time PCR Detection System with each well containing the following conditions: 10 μL Sso FastTM EvaGreen® Supermix (Bio-Rad, Canada), 5.0 μL of appropriately diluted genomic DNA, 1.0 μL of each primer (10 μM) (Table 1) and 3.0 μL of double distilled water with a total well volume of 20 μL. RT-PCR cycling was 120 seconds at 98°C followed by 40 cycles of 5 seconds at 98°C and 5 seconds at 58°C. Three technical replicates were tested for each transformant to ensure consistency and accuracy. To ensure specificity of primers, melt curves were produced for each RT-PCR experiment. All primers were shown to amplify specific sequences and showed only one melting temperature on the melting curve. Serial dilutions of genomic DNA and a temperature gradient were used in RT-PCR in order to determine the efficiencies of all reactions and were found to be between 90-110% efficient. *Tef1a* Real-Time primers were used for the reference gene and data were normalized using tef1α primers.

### Inoculum preparation and β-glucosidase production

After 14 days of incubation at 30°C, the greenish conidia from engineered *T. reesei* carrying pPtef1-*bgl1-cbh1* were suspended in 5 mL of sterile saline solution (0.9% w/v, NaCl). The spores were separated from the mycelia by gentle filtration through 12 layers of lens paper (Fisher Scientific, Canada), and spores were counted using a Petroff-Hausser cell counter (American Optical, USA). The isolated spores were added at 1.0 × 107 (final concentration) to 250 mL flasks containing 50 mL medium (MA-medium) with 1% glucose (w/v) as the carbon source and incubated at 30°C for a total of 24 hours. Pre-grown mycelia were washed three times by MA-medium with no carbon source to remove any residual glucose. The mycelia were then transferred into 250 mL flasks containing 50 mL cellulase-inducing medium (MA-medium) in which 1% glucose (w/v) was substituted with 1% microcrystalline cellulose (w/v) [37]. Three biological replicates were done for each transformant. To examine β-glucosidase activity and total cellulase activity, a time course trial was conducted similar to that of Cianchetta et al. [38]. Specifically, the flasks were incubated at 30°C for a total of 144 hours. Samples of 500 μL each were taken from each of the flasks every 24 h. These 500 μL samples were centrifuged at 16060 rcf for 5 min, and the supernatant was used as the source of enzyme [30].

### Enzyme assays

Fermentation broth was centrifuged, and aliquots of the supernatant were diluted to assay the enzyme activities. All enzyme activities were expressed as specific activities using international units per mg protein in the supernatant (one unit corresponds to the amount of enzyme required to liberate 1 μmol of product per minute under the standard assay conditions).The protein concentration in the supernatant was measured using the Fermentas Bradford Reagent and also Fermentas bovine serum albumin (BSA) standard set as the standard (Fermentas, Canada).

β-glucosidase activity was determined according to method described by Korotkova using the initial rate of the accumulation of the colored reaction product [39]. Briefly, 20 μL of diluted enzyme (culture supernatant) was added into each microplate well (pre-heated at 70°C for 10 min) containing 180 μL of 5 mM *p*NPG in 50 mM sodium citrate buffer, pH 5.0 as the substrate. The plate was incubated at 70°C for 10 min before stopping the reaction by adding 100 μL of 1 M cold sodium carbonate. The release of *p*-nitrophenyl by enzymatic hydrolysis was indicated by the appearance of yellow color and monitored at 405 nm by xMark Microplate Spectrophotometer (Bio-Rad, Canada). The absorbance of the samples was normalized by the enzyme blanks (20 μL enzyme and 180 μL of the assay buffer) and the substrate blank (20 μL of the assay buffer and 180 μL of the substrate).

The optimal pH of BGLI activity was measured at pH ranging from 3.0 to 10.0 under the standard assay conditions at 70°C for 10 min. The buffers used in the experiment were 50 mM sodium citrate (pH 3.0-6.0), 50 mM sodium acetate (pH 4.0-6.0), 50 mM MOPS (pH 6.0-8.0), and 50 mM Tris (pH 8.0-10.0). The pH stability was analyzed by pre-incubating 10 μL of BGLI in 90 μL of buffers mentioned above at 25°C for 4 h. Of this, 20 μL of the enzyme mixture was then used to determine remaining activity at 70°C in sodium citrate buffer, pH 5.0, for 10 min [15].

The optimal temperature of BGLI activity was determined by incubating the enzyme (aliquots of supernatant) at different temperatures ranging from 20 to 90°C in 50 mM sodium citrate pH 5.0 for 10 min. The thermostability of the enzyme was analyzed by measuring the residual activity at the optimal conditions (70°C and 50 mM sodium citrate buffer, pH 5.0, for 10 min) after pre-incubating the enzyme at 30–90°C for 30–120 min. Relative activity was calculated as enzymatic activity at the indicated temperature divided by the maximal activity at the optimal temperature [15].

For detection of in gel β-glucosidase activity, samples were analyzed by native PAGE using 8% and 5% polyacrylamide as separation and stacking gels, respectively. Electrophoresis was run at a constant current of 25 mA at 4°C for 5 h using Hoefer SE 600 Ruby (Amersham Biosciences, USA). Gel was washed with distilled water and overlaid with 5 mM 4- methylumbelliferyl β-D-glucopyranoside (MUG, Sigma-Aldrich, Canada) in 50 mM sodium citrate buffer (pH 5.0) and incubated at 70°C for 10 min. The presence of fluorescent reaction product was visualized under UV light using gel documentation system (Syngene, Canada).

Total cellulase activity was measured using microplate based filter paper assay according to a method described previously [37], which is 25-fold scale-down of the International Union of Pure and Applied Chemistry (IUPAC) protocol for FPA assay [40-42]. In the assay, 6 mm diameter filter paper disk (Whatman No. 1, with average weight of 3.0 mg each, ThermoFisher Scientific, Canada) in 75 mM citrate buffer (pH 4.8) was used as the substrate. The reducing sugars released were measured using 3,5-dinitrosalicylic acid (DNS reagent) with the absorbance measured at 540 nm. The substrate control (containing only the filter paper, and the buffer) and enzyme control (containing enzyme and the buffer with no filter paper) were also tested and subtracted from the absorbance. Total reducing sugars generated during the assay was estimated as glucose equivalents. Filter paper unit (FPU/mL) was first calculated using the equationpreviously described by Xiao et al. [42] and then was converted to FPU/mg using the protein concentration accordingly. One FPU is defined as an average of one μmole of glucose equivalents released per min in the assay reaction.

Endoglucanase activity (EG) was measured using 2% (w/v) carboxymethylcellulose (CMC) in citrate buffer (50 mM, pH 4.8) as the substrate according to the method described by Zhang et al. [43]. The enzymes were added to the substrate solution (pre-equilibrated at 50°C) and incubated at 50°C for 30 min. Glucose released was measured by DNS method at 540 nm and using a glucose standard curve after deduction of the enzyme blank absorbance. Exoglucanase activity (Exo) was measured using 1.25% (w/v) Avicel (PH 105) in sodium acetate buffer (0.1 M, pH 4.8) as the substrate according to the method described by Zhang et al. [43]. The enzymes were added to the substrate solution (pre-equilibrated at 50°C) and incubated at 50°C for 2 h. The total soluble sugars released in the assay were determined using phenol (5%)- sulfuric acid (98%) assay at 490 nm. The enzyme activity was calculated on the basis of a linear relationship between the total soluble sugar released and enzyme dilution [43]. One unit of exoglucanase activity is defined as the amount of enzyme that releases one micromole of glucose equivalent per minute from Avicel.

### Enzymatic hydrolysis of biomass by the BGLI-overexpressing *T. Reesei* transformants

In order to evaluate hydrolysis activity of the engineered *T. reesei* with enhanced β- glucosidase activity, either 3% (w/v) of barley straw (untreated, Organosolv- or NaOH- pretreated) or 3% of microcrystalline cellulose were used according to method described by Cheng et al. [44]. Substrate hydrolysis was catalyzed by the culture supernatants collected as described above. Experiments were started with 3% substrate concentration in 750 μl buffer (50 mM sodium citrate buffer at pH 5.0 with 1 mM sodium azide to prevent microbial contamination) and 750 μl crude enzyme dosage at 50°C for 72 h. For a control sample, the crude enzyme was replaced with the buffer. Samples were taken every 24 h and subjected to determination of the glucose and reducing sugar levels in the supernatant. The reducing sugars were detected by DNS method and the glucose concentration was measured using glucose oxidase assay kit (QuantiChrom™ Glucose Assay Kit, Medicorp, Canada).

### Data processing and statistical analysis

All experimental points are the average values of three independent experiments. The data was collected in a Microsoft Excel spreadsheet where the average and standard error of the mean were determined. The graphs were created using the software PRISM 5.0. A one-way analysis of variance (one-way ANOVA) at a confidence level of 99% (α = 0.01) was carried out with the software PRISM 5 to test the statistical significance of differences between the Exoglucanases as well as Endoglucanases activities of the four *T. reesei* transformant strains (T1-T4) compared to the parental *T. reesei* strain (Figure 3C).

## Abbreviations

BCC, BIOTEC Culture Collection; BGL, β-glucosidases; Bgl, β-glucosidases gene; cbh1, Cellobiohydrolases I; CMC, Carboxymethylcellulose; cP, Periconia sp. cDNA; DNS, 3,5-dinitrosalicylic acid; EG, Endoglucanases; Exo, Exoglucanases; FPA, Filter paper assay; FPU, Filter paper unit; gP, Periconia sp. genomic DNA; G, Glucose; gpd1, Glyceraldehyde-3-phosphate dehydrogenase gene; hph, Hygromycin B phosphotransferase; MA, Mandel and Andreotti; MUG, 4-methylumbelliferyl β-D-glucopyranoside; P, Periconia sp. strain; PAGE, Polyacrylamide gel electrophoresis; PDA, Potato dextrose agar; Pgpd1, gpd1 promoter; pNPG, p-nitrophenyl-β-D-glucopyranoside; Ptef1, tef1 promoter; QM, T. reesei QM9414 strain; qRT-PCR, Quantitative Real-Time PCR; RS, Reducing sugars; SDS-PAGE, Sodium dodecyl sulfate polyacrylamide gel electrophoresis; SR, Solid residues; T, Transformants strain; Tcbh1, cbh1 terminator; tef1α, Translation elongation factor 1-alpha gene; Tgpd1, gpd1 terminator.

## Competing interests

The authors declare that they have no competing interests.

## Authors’ contributions

MD designed, carried out the experiments and prepared the manuscript. WQ coordinated and supervised the research as well as contributed to the manuscript editing. All authors read and approved the final version of the manuscript.
